# Clinical application of a novel rib fracture score system in patients with multiple rib fractures

**DOI:** 10.3389/fsurg.2025.1653221

**Published:** 2026-01-08

**Authors:** Jianpeng Zhang, Weiqiang Li, Zhidong Liu

**Affiliations:** 1 Second Department of Thoracic Surgery, Beijing Chest Hospital, Capital Medical University, Beijing, China; 2 Department of Thoracic Surgery, Beijing Luhe Hospital, Capital Medical University, Beijing, China

**Keywords:** thoracic injuries, rib fractures, fracture fixation, prognosis, pneumonia

## Abstract

**Objectives:**

To validate a new type of rib fracture score system for treatment decisions in patients with multiple rib fractures.

**Methods:**

This retrospective study included patients admitted with multiple rib fractures from one clinical center between April 2017 and April 2022. Patients were divided into the conservative group and the surgical group based on their management strategy. The patients were assessed using a novel rib fracture score system based on anatomical and pathophysiological characteristics.

**Results:**

In a study of 564 patients, 290 underwent surgical treatment while 274 received conservative treatment. Surgical patients had significantly higher satisfaction scores (*P* < 0.001) and a greater prevalence of chronic diseases (*P* = 0.022). The optimal satisfaction cut-off was 9.5 points, dividing patients into low score (LS, <10) and high score (HS, ≥10) groups. In the LS group, pneumonia incidence was similar (*P* > 0.05). However, in the HS group, surgical patients experienced lower pneumonia rates (no pneumonia: 76.1% vs. 11.5%, *P* < 0.001), reduced ventilator hours (8.1 ± 37.6 vs. 23.7 ± 66.7, *P* < 0.001), and less opioid use (1.2 ± 1.5 vs. 2.7 ± 2.5 times, *P* < 0.001). For scores ≥15, conservative treatment may lead to increased respiratory complications.

**Conclusions:**

Clinical application of the novel rib fracture score system may be efficient in stratifying patients with rib fractures. Patients with a score ≥10 may benefit from surgical intervention. Patients with a score ≥15 may have a greater risk of respiratory complications during the period of conservative treatment compared with the LS group, suggesting the importance of surgical intervention in such patients.

## Introduction

Rib fractures are often seen in blunt chest trauma (1). A conservative estimate suggests that the number of patients with rib fractures in China can reach 1.5–2 million per year (2). A reasonable and effective evaluation method is essential to assess the severity of rib fractures and help in management, especially considering that the surgical stabilization of rib fractures (SSRF) in patients with major trauma can have important prognostic implications (3).

Various scores for rib fracture assessment have been proposed and associated with morbidity and mortality risks, such as the Rib Fracture Score (RFS) (4, 5). Although the RFS is easy to calculate, it cannot fully reflect the severity of rib fractures, especially bilateral ones (1, 6). Another tool, the Rib Score (RS), is based on imaging (7). The RS is solely based on the anatomical changes of fractures and does not include other prognostic factors, such as pain and age. While the RS can predict respiratory complications, it is less predictive of long-term quality of life (8, 9).

Patients with multiple rib fractures often suffer from respiratory distress syndrome, ventilator-assisted respiration, or tracheotomy and do not have a fatal outcome but can develop severe acute and chronic pain (10, 11). Relying solely on anatomical changes or pain scores for surgical indications is not accurate enough (12). An ideal rib fracture scoring system should accurately describe the degree of anatomical changes in the fracture region, reflect the interaction between multiple rib fractures, and consider the patient's pathophysiological changes.

Therefore, this study validated a novel rib fracture score system that contains an anatomical position score and a pathophysiological score among patients with multiple rib fractures.

## Materials and methods

### Study population and setting

This retrospective study included patients admitted with multiple rib fractures between April 2017 and April 2022 at the Thoracic Surgery Department of Beijing Luhe Hospital affiliated to Capital Medical University.

The inclusion criteria were 1) between 18 and 80 years of age. 2) rib fractures with floating segments of the chest wall. Floating segments of the chest wall were defined as two transversely adjacent bicortical fractures of the same rib, or two consecutive longitudinal bicortical rib fractures constitute the basic unit of the floating segment of the chest wall.

The exclusion criteria were 1) patients with multiple injuries where any part, except the chest, is scored ≥3 points according to the AIS score (13), 2) patients with rib fracture combined with sternal fracture, severe pulmonary laceration, bronchial rupture, massive hemothorax, diaphragmatic rupture, or other serious thoracic organ injuries requiring emergency thoracic exploration, 3) patients with open chest wall fracture, 4) patients with concomitant bacteremia, chest wall infection, or chest cavity infection, 5) serious medical conditions such as recent new cerebral infarction, myocardial infarction, or hemorrhagic disease, 6) diseases or conditions that may cause a serious decline in the quality of life, including physical disability, severe sequelae of cerebral infarction, poorly controlled chronic obstructive pulmonary disease, or cardiac failure, 7) expression limitations or abnormalities or psychiatric disorders.

All patients received the following treatments, including: 1) analgesia. ① Patients took medications such as Lornoxicam, Dihydrocodeine Paracetamol, Nabumetone, and other as prescribed. ② Dihydrocodeine Hydrochloride (100 mg/vial) and Pethidine Hydrochloride Injection (50 mg/vial) were administered as needed. 2) Management of pneumothorax and hemothorax. In accordance with the routine of thoracic surgery and based on the specific situation of each patient, thoracic drainage was performed for patients with pneumothorax with lung compression over 30% and patients with moderate or more hemothorax, with drainage tube diameter ranging from 16Fr to 24Fr.

During the treatment process, the professional care team communicated the patient's condition and treatment options. Patients who underwent surgery needed to meet at least one of the following criteria [3]: 1) a radiographic flail segment, 2) ≥5 consecutive rib fractures, 3) ≥3 displaced rib fractures. Patients decided whether to opt for surgical or conservative treatment based on the information provided. The team closely monitored changes in each patient's condition, ensuring their medical safety and best interests.

This study was approved by the Ethics Committee of Beijing Luhe Hospital affiliated to Capital Medical University (approval #2023-LHYY-010-02; project #LHYY2023-YJZ010). Informed consent was waived by the committee due to the retrospective nature of the study. The STROBE guideline was used to ensure proper reporting of methods, results, and discussion.

### Outcomes and data collection

General information was collected for all patients, including age, sex, comorbidities, novel rib fracture score, management of comorbidities, tracheal intubation or incision, intensive care unit (ICU) time, and ventilator use, pneumonia, lower extremity deep vein thrombosis, frequency of opioid analgesic medication, total hospitalization time, numerical rating scale (NRS) (14), and the SF-12 Quality of Life Survey score (15). For patients in the surgical group, the NRS was assessed at 24 h, 72 h, 2 weeks, 4 weeks, 6 weeks, 3 months, 6 months, and 9 months postoperatively, and the SF-12 was assessed at 3, 6, and 9 months postoperatively. For patients in the conservative group, the NRS was evaluated at 1 week, 2 weeks, 4 weeks, 6 weeks, 3 months, 6 months, and 9 months post-injury, and the SF-12 at 3 months, 6 months, and 9 months post-injury. At the end of the main treatment, the patient's costs, time to return to work or previous life, and satisfaction with the treatment received were collected.

### Novel score

The novel score is the sum of the anatomical score and the pathophysiological score, inspired by two previous studies (16, 17) that were still deemed insufficient by the authors to grasp the actual situation of the patients.

In the anatomical scoring section, scoring was based on the floating segment we proposed, which distinguishes it from the flail segment that has appeared in previous studies (17). The essence of the floating segment is the number of bicortical rib fractures within a certain range, which encompasses the following implications: (1) only bicortical fractures were included in the count (6). (2) It must be consecutive ribs. If a patient has a fracture of ribs 3–7 and a fracture of rib 9, the fracture of rib 9 is considered an isolated fracture. It is not counted in the scoring because it is not strongly associated with the region of the main fracture. (3) All rib fractures should be close to each other, and the furthest transverse distance between them should be less than half of the transverse distance of the measurements described later, and fractures beyond the region are not included in the scoring. This study aimed to develop a descriptive method for analyzing rib fractures in patients, emphasizing the interplay between fractures that are in close proximity to one another. It is important to note that this focus does not imply that isolated fractures lack a significant negative effect on patient outcomes. This scoring approach is a hypothesis that we have proposed based on several considerations: (1) Bicortical rib fractures affect patients more than monocortical fractures (6). (2) Floating segments emphasize the linkage effect of rib fractures on each other within a certain range. Some studies have shown a lower incidence of pneumonia in patients with isolated rib fractures alone (18). Therefore, we considered fractures that were out of range or had a break in continuity as isolated fractures and were not counted in the scoring. It can be seen that the anatomical score is a description and evaluation of the main part of the patient's rib fracture, which is closer to the clinic practice because the main part of the patient's rib fracture is usually the extent of the surgically fixed rib fracture.

Six items were considered for the anatomical score (Supplementary Figure S1). 1) Two rib fractures are projected perpendicularly to the contiguous anterior and posterior medians and the distances between the two projected points are documented as the transverse distance. 2) Transverse floating segment counts: at least two bicortical fractures occurring in the same rib of the patient, and the transverse distance between the two fractures is equal to or shorter than half of the distance between the anterior and posterior midline of the patient's chest wall. All bicortical fractures within the range of the same rib are counted, the number of counts is at least 2, and the number of fractures beyond the range is counted as 0. 3) Longitudinal floating segment counts: longitudinally aligned rib fractures need to be consecutive. The furthest transverse distances of the longitudinal fracture need to be less than half the distance from the anterior-posterior midline of the patient's chest wall. Rib fractures outside the range are isolated and have an interruption of continuity. Interrupted isolated fractures are counted as 0. Bicortical rib fractures that form a floating segment again after the interruption are counted along with the previous one. The count is at least 2. 4) If a patient has a bicortical rib fracture within a floating segment and the fracture break is displaced more than half the distance between the bicortical layers of the rib at that location (19), the fracture count at that location is 2; otherwise, it is 1. The cortical spacing of the rib bilayers needs to be measured in the transverse axial position on chest computed tomography (CT). 5) Rib cartilage fracture was treated as a bicortical rib fracture and counted in the same way as before. 6) Rib fractures where there is overlap between the transverse floating segment and the longitudinal floating segment count are not repeated. The sum of the transverse floating segment count and the longitudinal floating segment count is the anatomical location score of the chest wall floating segment count for that patient.

In addition to the anatomical location score of floating segments, the novel score had a pathophysiological component (Supplementary Table S1). Patients with the following factors were scored cumulatively according to the scoring requirements. 1) Pain is an important factor (3, 20, 21). Patients with the highest NRS of 5–6 at the time of injury to the time of consultation were scored 0; 7–8 were scored 1; 9–10 were scored 2; patients with persistent mandatory protective positions (e.g., mandatory seated, flat, or side-lying) were scored 3, disregarding the NRS. 2) Patients with a combination of hemorrhagic (and/or pneumothorax) (22, 23) were scored 1. 3) The Wagner pulmonary contusion score was used to assess the severity of pulmonary contusion (24). The Wagner score is based on CT imaging. It classifies the severity of injury into three levels according to the percentage of lung injury in the total lung volume: ≤18% of the lung injury is classified as grade I, 19% to 27% is classified as grade II, and ≥28% is classified as grade III, which are scored as 1, 2, and 3 points, respectively. 4) The patient's oxygenation status can influence prognosis (25, 26). One point is scored for a patient presenting with symptoms of cough and sputum; 2 points for a patient with unfavorable cough and sputum as judged by a specialist but not yet resulting in the latter condition; 3 points for a patient with a partial pressure of oxygen ≤65 mmHg on non-oxygen inhalation; 3 points for a patient with a progressive decrease in finger pulse oximetry or a sustained decrease below 90%. 5) Patient respiratory rate of ≥21 times/min is scored 1 point; ≥25 times/min, 2 points. 6) Age ≥60 years old is scored 1 point, ≥70 years old, 2 points (27).

The final patient's novel score was the sum of the anatomical score plus the pathophysiological score. For patients with bilateral floating segments, the scores were based on anatomical scores for both sides plus the patient's individual pathophysiological score.

Clinical pneumonia was an outcome event based on 1) chest x-ray or CT showing emerging or progressive infiltrative, solid, or ground glass shadows, and 2) plus two or more among i) fever with temperature >38 °C, ii) purulent airway secretions, iii) peripheral blood leukocyte count >10 × 10^9^/L or <4 × 10^9^/L, and iv) culture of pathogenic bacteria in qualified lower respiratory secretions and bronchial lavage fluid, consistent with the clinical manifestations (28, 29).

### Statistical methods

SPSS 24.0 (IBM Corp., New York, USA) was used for statistical analysis. The categorical data were described as *n* (%) and compared with the chi-squared test or Fisher's exact test (when T < 5 or *n* < 40). The continuous data were described using means ± standard deviations (mean ± SD). Continuous data not meeting the normal distribution were analyzed using the Wilcoxon rank sum test. Subgroup analysis was performed based on whether pneumonia occurred. Receiver operating characteristic (ROC) curves were used to analyze the relationship between the novel score and patient satisfaction. The point with the optimal discriminative effect was selected as the best cut-off value of the novel score, and patients were stratified into two groups according to the cut-off value. Observational indicators were compared based on the novel score stratification. Two-sided *P* < 0.05 was defined as a statistically significant difference.

## Results

### Characteristics of the patients

**Figure 1:**
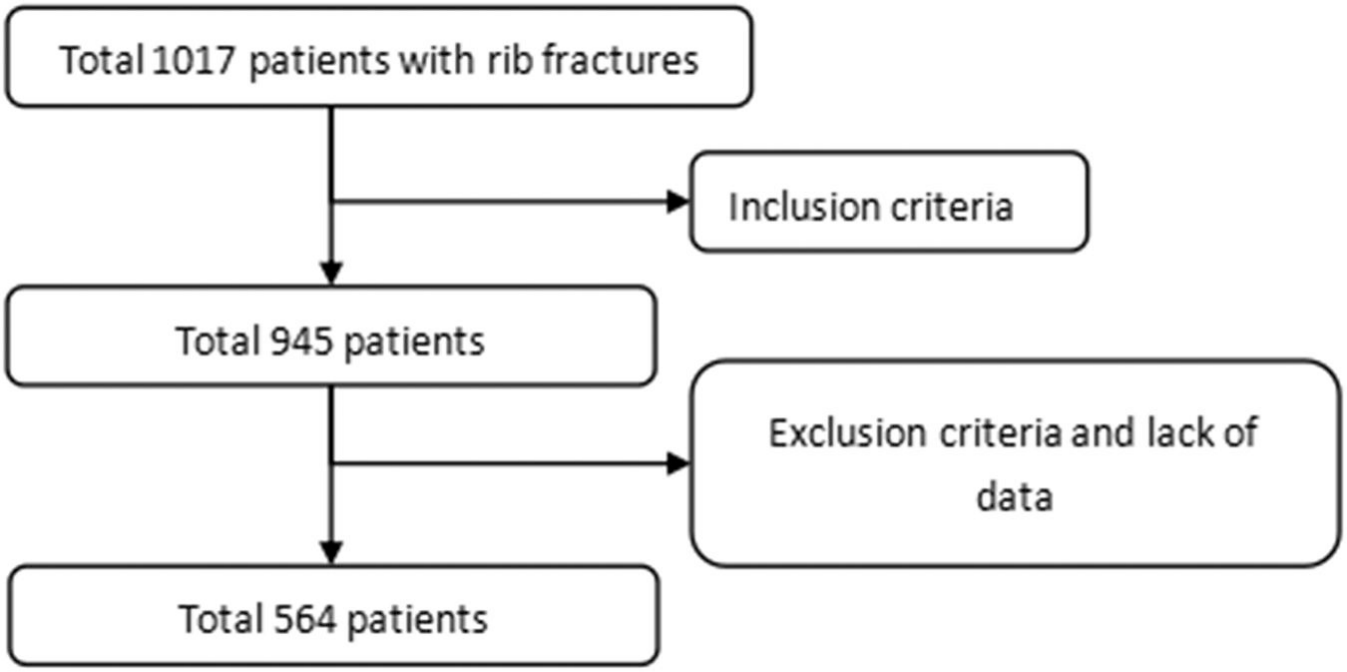
Flow diagram of the study.

During the study period, a total of 1,017 patients with rib fractures were admitted. Of these, 945 patients met the inclusion criteria and were enrolled in the study. A total of 305 patients were excluded based on the following criteria: 191 patients were excluded due to criterion 1, 54 due to criterion 2, 14 due to criterion 3, 7 due to criterion 4, 17 due to criterion 5, 15 due to criterion 6, and 7 due to criterion 7. Additionally, 76 patients were excluded due to missing information. Ultimately, 564 patients were included in the analysis, comprising 274 in the conservative treatment group and 290 in the surgical treatment group (Figure 1). None of the patients died during follow-up.

**Figure 2:**
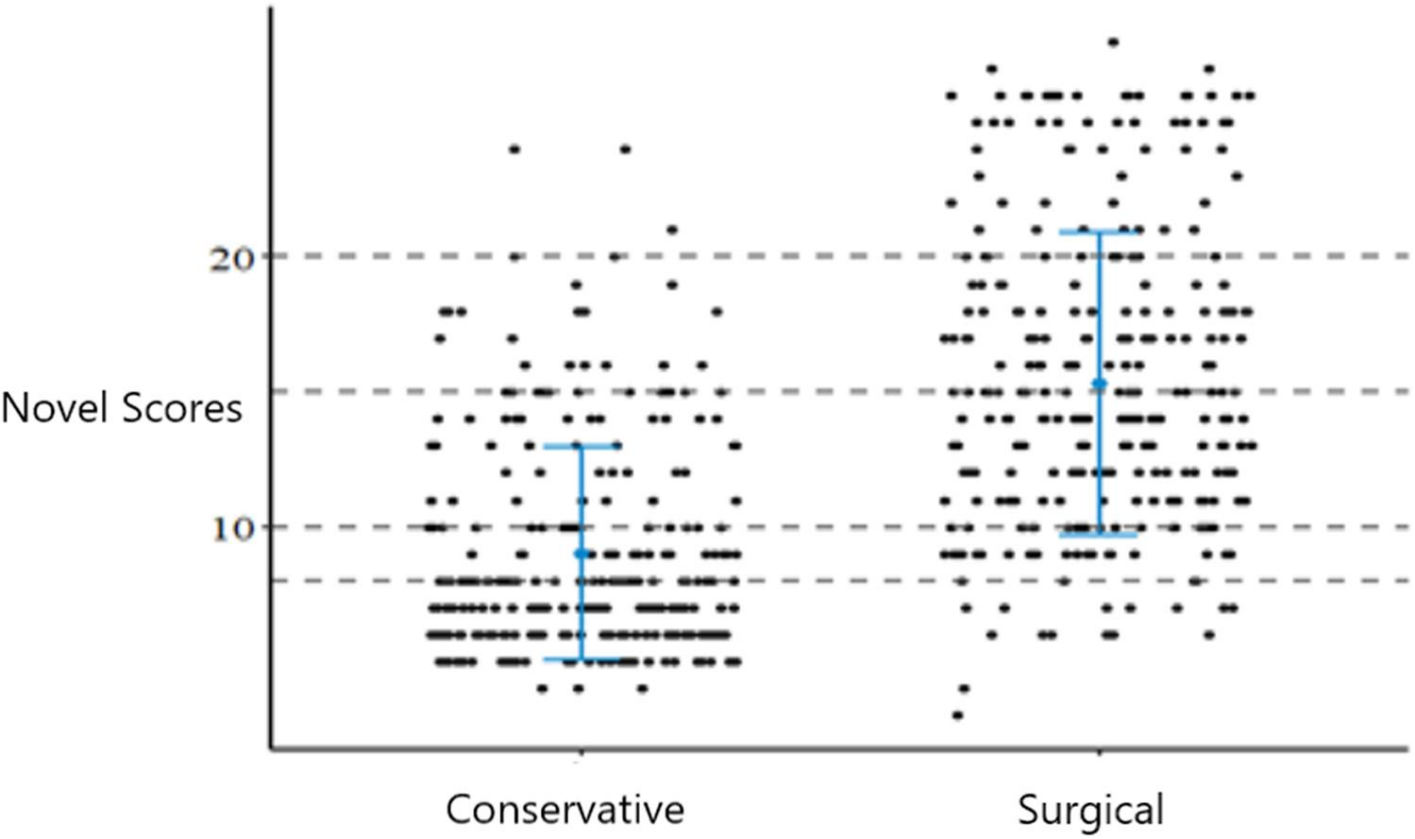
Scatterplot distribution of novel scores for the two groups.

There were no significant differences in age (*P* = 0.091), sex (*P* = 0.243), and fracture site (*P* = 0.656) between the two groups. The proportion of patients with comorbidities in the surgical group was significantly higher than in the conservative group (35.2% vs. 25.9%, *P* = 0.022). The novel score was significantly higher in the surgical group (15.3 ± 5.6 vs. 9.0 ± 3.9, *P* < 0.001) (Table 1). The distribution of the novel scores is shown in Figure 2.

**Table 1:** Characterization and comparison of conservative and surgical groups.

Items	Surgical group (*n* = 290)	Conservative group (*n* = 274)	*P*
Age, year	51.8 ± 10.9	53.6 ± 14.3	0.091
Gender, *N* (%)			0.243
Male	221 (76.2%)	196 (71.5%)	
Female	69 (23.8%)	78 (28.5%)	
Novel score	15.3 ± 5.6	9.0 ± 3.9	<0.001
Combined chronic diseases, *N* (%)			0.022
Yes	102 (35.2%)	71 (25.9%)	
No	188 (64.8%)	203 (74.1%)	
Rib fracture site, *N* (%)			0.656
Left	113 (39.0%)	117 (42.7%)	
Right	110 (37.9%)	99 (36.1%)	
Bilateral	67 (23.1%)	58 (21.2%)	

### Level of satisfaction of the patients

**Figure 3:**
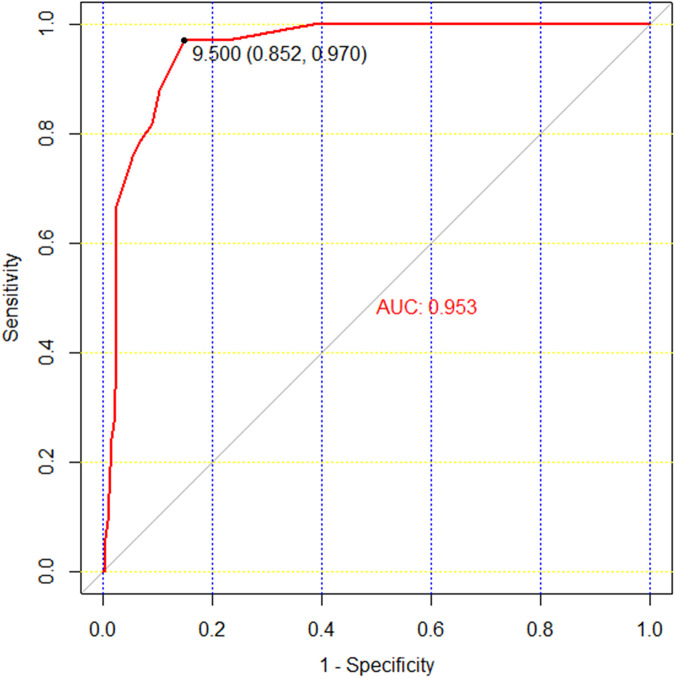
Novel score cut-off values for treatment satisfaction in the conservative group.

Upon follow-up completion, the treatment team assessed patient regret regarding received therapies. In the surgical group, all 290 patients reported no regret regarding their surgical intervention. Conversely, in the conservative group, 38 patients (38/274) were ambivalent about their satisfaction with non-operative care. Furthermore, 33 patients (33/274) expressed regret over their conservative approach, favoring surgical intervention. The conservative group's treatment acceptance rate was 74.1% (203/274).The relationship between treatment satisfaction and the novel score of patients in the conservative group showed that the optimal cut-off point was at 9.5 points (ROC AUC = 0.953) (Figure 3). Since the scoring system is based on whole numbers, the rounded cut-off score (10 points) was used to stratify patients into two groups: patients with novel scores <10 vs. ≥10.

### Characteristics of the patients based on the novel score

After stratification, there were 43 patients who underwent surgical treatment and 187 who received conservative treatment in the score <10 group. The patients who underwent surgical treatment had longer hospitalization (9.9 ± 2.8 vs. 3.3 ± 7.6 days, *P* < 0.001) but shorter time to return to work/life (9.4 ± 1.5 vs. 12.4 ± 2.9 weeks, *P* < 0.001). There were significant differences in the cost bearer (*P* < 0.001), and a higher proportion of patients who received surgical treatment had insurance (67.4% vs. 33.7%) (Table 2).

**Table 2:** Comparison of conservative and surgical group information based on novel score matching.

Items	Novel score <10			Novel score ≥ 10		
	Surgical (*n* = 43)	Conservative (*n* = 187)	*P*	Surgical (*n* = 247)	Conservative (*n* = 87)	*P*
Age, year	49.4 ± 11.6	51.6 ± 13.6	0.367	52.3 ± 10.8	58.1 ± 14.9	**<0** **.** **001**
Gender, *N* (%)			0.327			0.311
Male	35 (81.4%)	136 (72.7%)		186 (75.3%)	60 (69.0%)	
Female	8 (18.6%)	51 (27.3%)		61 (24.7%)	27 (31.0%)	
Combined chronic diseases, *N* (%)			0.079			0.896
Yes	15 (34.9%)	39 (20.9%)		87 (35.2%)	32 (36.8%)	
No	28 (65.1%)	148 (79.1%)		160 (64.8%)	55 (63.2%)	
Hospitalization, day	9.9 ± 2.8	3.3 ± 7.6	**<0**.**001**	16.7 ± 17.4	14.9 ± 14.3	0.349
Return to work/life time, week	9.4 ± 1.5	12.4 ± 2.9	**<0**.**001**	11.5 ± 1.7	16.9 ± 3.8	**<0**.**001**
Cost Bearer, *N* (%)			**<0**.**001**			0.078
Self-financed	1 (2.3%)	21 (11.2%)		2 (0.8%)	2 (2.3%)	
Insurance	29 (67.4%)	63 (33.7%)		54 (21.9%)	11 (12.6%)	
Others assume	13 (30.2%)	103 (55.1%)		191 (77.3%)	74 (85.1%)	
Repentance, *N* (%)		0.243			**<0.001**	
No	43 (100%)	173 (92.5%)		247 (100%)	30 (34.4%)	
Yes	0 (0%)	1 (0.5%)		0 (0%)	32 (36.8%)	
Inconclusive	0 (0%)	13 (7.0%)		0 (0%)	25 (28.7%)	
Serious complication						
Pneumonia, *N* (%)			0.589			**<0**.**001**
No	43 (100%)	173 (92.5%)		188 (76.1%)	10 (11.5%)	
Left	0 (0%)	4 (2.1%)		26 (10.5%)	28 (32.2%)	
Right	0 (0%)	7 (3.7%)		19 (7.7%)	29 (33.3%)	
Bilateral	0 (0%)	3 (1.6%)		14 (5.7%)	20 (23.0%)	
Tracheal Intubation, *N* (%)			0.566			0.352
Yes	1 (2.3%)	3 (1.6%)		29 (11.7%)	14 (16.1%)	
No	42 (97.7%)	184 (98.4%)		218 (88.3%)	73 (83.9%)	
Tracheotomy, *N* (%)			1			0.167
Yes	0 (0%)	0 (0%)		1 (0.4%)	2 (2.3%)	
No	43 (100%)	187 (100%)		246 (99.6%)	85 (97.7%)	
Deep vein thrombosis, *N* (%)			1			0.422
No	43 (100%)	185 (98.9%)		229 (92.7%)	78 (89.7%)	
Left	0 (0%)	1 (0.5%)		4 (1.6%)	3 (3.4%)	
Right	0 (0%)	0 (0%)		6 (2.4%)	1 (1.1%)	
Bilateral	0 (0%)	1 (0.5%)		8 (3.2%)	5 (5.7%)	
Ventilator use, hour	0.0 ± 0.0	1.1 ± 13.0	0.408	8.1 ± 37.6	23.7 ± 66.7	**0**.**018**
ICU Duration, hour	0.5 ± 3.4	1.4 ± 16.6	0.745	16.1 ± 63.5	28.3 ± 78.7	0.3
Opioid use, times	0.3 ± 1.1	0.5 ± 1.3	0.405	1.2 ± 1.5	2.7 ± 2.5	**<0**.**001**

Items in bold font indicate statistically significant differences.

Among the 334 patients with a novel score of ≥10 points, 247 and 87 patients underwent surgical and conservative treatments, respectively. The patients who underwent surgery were younger (52.3 ± 10.8 vs. 58.1 ± 14.9, *P* < 0.001), with a shorter return to work/life time (11.5 ± 1.7 vs. 16.9 ± 3.8 weeks, *P* < 0.001), less pneumonia rate (no pneumonia: 76.1% vs. 11.5%, *P* < 0.001), less ventilator usage (8.1 ± 37.6 vs. 23.7 ± 66.7 h, *P* < 0.001), and less opioid use (1.2 ± 1.5 vs. 2.7 ± 2.5 times, *P* < 0.001) (Table 2).

### NRS and quality of life

A total of 277 patients in the surgical group and 263 in the conservative group completed the NRS evaluation. Results showed that except for the 9-month time point, all patients who received conservative treatments had a higher NRS (all *P* < 0.05) (Table 3).

**Table 3:** Comparison of pain scores at different time points in the surgical and conservative group.

Point	All patients			Novel score <10			Novel score ≥ 10		
	Surgical (*n* = 277)	Conservative (*n* = 263)	*P*	Surgical (*n* = 43)	Conservative (*n* = 184)	*P*	Surgical (*n* = 234)	Conservative (*n* = 79)	*P*
72 h or 1 week after treatment	3.54 ± 0.61	5.03 ± 1.03	<0.001	3.02 ± 0.46	4.62 ± 0.78	<0.001	3.64 ± 0.59	6.00 ± 0.88	<0.001
2 weeks after treatment	2.76 ± 0.63	4.20 ± 0.82	<0.001	2.40 ± 0.49	3.88 ± 0.63	<0.001	2.82 ± 0.63	4.95 ± 0.72	<0.001
4 weeks after treatment	2.49 ± 0.58	3.54 ± 0.81	<0.001	2.09 ± 0.29	3.25 ± 0.67	<0.001	2.56 ± 0.59	4.20 ± 0.69	<0.001
6 weeks after treatment	1.72 ± 0.57	2.73 ± 0.92	<0.001	1.30 ± 0.46	2.39 ± 0.72	<0.001	1.79 ± 0.56	3.48 ± 0.86	<0.001
3 months after treatment	0.47 ± 0.62	1.18 ± 0.85	<0.001	0.00 ± 0.00	0.85 ± 0.65	<0.001	0.55 ± 0.64	1.94 ± 0.78	<0.001
6 months after treatment	0.23 ± 0.45	0.51 ± 0.67	<0.001	0.00 ± 0.00	0.24 ± 0.47	0.001	0.27 ± 0.47	1.11 ± 0.69	<0.001
9 months after treatment	0.05 ± 0.26	0.12 ± 0.35	0.002	0.00 ± 0.00	0.01 ± 0.10	0.499	0.06 ± 0.28	0.37 ± 0.53	<0.001

The SF-12 was completed by 168 patients in the surgical group and 182 in the conservative group. The SF-12 quality of life scores of the patients in the surgery group were higher than those of the patients in the conservative group at 3, 6, and 9 months (all *P* < 0.05), except for patients with novel score <10 at 9 months after treatment, among whom no statistically significant difference on SF-12-MCS score was observed (52.54 ± 1.22 vs. 52.36 ± 2.05, *P* = 0.450) (Table 4).

**Table 4:** Comparison of SF-12 scores at different time points in the surgical and conservative groups.

Point	All patients			Novel score <10			Novel score ≥ 10		
	Surgical (*n* = 168)	Conservative (*n* = 182)	*P*	Surgical (*n* = 37)	Conservative (*n* = 141)	*P*	Surgical (*n* = 131)	Conservative (*n* = 41)	*P*
SF-12-PCS									
3 months after treatment	50.22 ± 2.17	41.36 ± 6.11	<0.001	51.14 ± 1.70	43.08 ± 5.27	<0.001	49.95 ± 2.22	35.45 ± 5.05	<0.001
6 months after treatment	52.00 ± 1.84	47.96 ± 5.81	<0.001	52.85 ± 1.56	49.51 ± 3.81	<0.001	51.76 ± 1.85	42.61 ± 8.00	<0.001
9 months after treatment	52.53 ± 1.56	51.38 ± 2.90	<0.001	53.04 ± 1.45	52.12 ± 2.30	0.049	52.39 ± 1.56	48.85 ± 3.32	<0.001
SF-12-MCS									
3 months after treatment	49.68 ± 2.17	44.99 ± 4.64	<0.001	50.27 ± 2.23	46.08 ± 3.99	<0.001	49.52 ± 2.13	41.26 ± 4.81	<0.001
6 months after treatment	51.40 ± 1.89	49.20 ± 3.81	<0.001	51.75 ± 1.72	50.35 ± 2.86	0.002	51.30 ± 1.93	45.28 ± 4.08	<0.001
9 months after treatment	52.26 ± 1.49	51.71 ± 2.89	0.04	52.54 ± 1.22	52.36 ± 2.05	0.45	52.19 ± 1.55	49.44 ± 4.04	<0.001

### Multivariable analysis for the development of pneumonia in all patients

Among the 564 patients, 414 did not develop pneumonia. The multivariable logistic regression analysis showed that only higher age (OR = 1.151, 95%CI: 1.017, 1.303, *P* = 0.026) and longer hospitalization (OR = 1.269, 95%CI: 1.023, 1.573, *P* = 0.030) showed significant results **(**Supplementary Table S2).

### Multivariable analysis for postoperative pneumonia in surgical patients

Among 290 patients who received surgical treatment, 30 patients experienced pneumonia postoperatively, including 27 who did not have pneumonia before surgery. Compared with patients without postoperative pneumonia, patients who experienced pneumonia postoperatively showed higher scores (22.1 ± 4.6 vs. 14.5 ± 5.2, *P* < 0.001). The multivariable logistic regression analysis showed that only higher age (OR = 1.151, 95%CI: 1.017, 1.303, *P* = 0.026) showed significant results (Supplementary Table S3).

### Subgroup analysis

Among all patients, 290 patients were classified into subgroups according to the duration of preoperative conservative treatment; the patients who received >70 h (which was the median) of conservative treatment showed a higher prevalence of deep vein thrombosis, preoperative pneumonia, combination of chronic disease, and longer hospital stay (Supplementary Table S4).

The subgroup analysis based on the number of fixed/total rib fractures showed that in patients with a number of fixed/total rib fractures ≤0.75 (the median), the novel score was significantly higher, and the prevalence of tracheal intubation and deep vein thrombosis was also higher (Supplementary Table S5). The subgroup analysis based on the number of fixed sites/total number of rib fracture sites showed similar results. The patients with a number of fixed sites/total number of rib fracture sites ≤0.75 (the median) had higher novel scores and prevalence of tracheal intubation and deep vein thrombosis (Supplementary Table S6).

## Discussion

While patient satisfaction with previous treatments may be influenced by numerous factors, the subjective experiences of patients, as the recipients of care, warrant attention. Their contentment and dissatisfaction with therapeutic approaches merit contemplation by medical professionals. However, previous studies have seldom delved into the subjective perceptions of patients (2, 3, 6, 11, 19, 20). This study validated a novel rib fracture score system and used results of patient satisfaction for cut-off point definition. The patients were categorized according to the novel score cut-off point, and the performance of different clinical characteristics under the scope of the novel score was evaluated. The results showed that the novel score has great potential for rib fracture assessment.

Currently, there is no specific and widely accepted scoring system for rib fracture analysis. The RFS, proposed by Easter (4), is calculated as RFS = B × S + A, where B stands for the number of fractures, S stands for whether the fracture occurred unilaterally (1 point) or bilaterally (2 points), and A stands for the age of the patient (0, 1, 2, 3, and 4 points for ages ≤50, 51–60, 61–70, 71–80, and ≥81, respectively). An RFS of >4 points is an independent risk factor for increased mortality (5). Although the RFS is easy to calculate, it cannot fully reflect the severity of rib fractures, and it does not consider the degree of fracture displacement, which can affect patient prognosis (1, 6). Hence, the RFS has a poor predictive ability for whether a patient undergoes rib fracture fixation (30). In addition, in patients with bilateral rib fractures, multiplying the total number of rib fractures by 2 would inevitably overestimate the score. The RS, proposed by Chapman et al. (7), assigns a score of 1 to each for the presence of the indicators, and the RS score is the some of the scores: 1) six or more rib fractures, 2) bilateral rib fractures, 3) flail segment, 4) three or more fractures with severe displacement of the broken ends, 5) fracture of the first rib, and 6) at least one fracture in each of the three anatomic regions of the rib. The RS is based on the anatomical changes of the fractures as a scoring criterion and does not consider pain (2, 3) or age (12, 27). The RS has a predictive ability for near-term respiratory complications but cannot predict patients' long-term quality of life (8, 9). Buchholz et al. proposed the Revised Intensity Battle Score (RIBS) system for poor outcome prediction in patients with rib fractures; their results showed an AUC of 0.858 on internal datasets and also showed great performance in the external validation set (22, 31). For patients with rib fractures with a novel score <10, there were no significant differences between conservative and surgical treatments in terms of pneumonia, ventilator use time, tracheal intubation, tracheotomy, deep vein thrombosis in the lower extremities, or frequency of opioid use.

Respiratory-related complications are the most common complications in patients with rib fractures (32). In the present study, the patients with pneumonia compared with non-pneumonia patients showed differences in terms of tracheal intubation rate, tracheotomy rate, ventilator-assisted time, ICU stay, total hospitalization days, lower extremity deep vein thrombosis rate, and frequency of opioid use. Moreover, the results showed that the novel score of patients with pneumonia (15.4 ± 5.1) was significantly higher than that of patients without pneumonia (11.1 ± 5.6). The results showed that for patients with a novel score of ≥15, continuing conservative treatment may result in an increase in the incidence of respiratory-related complications, which may result in prolonged ICU stays and total hospitalization. Hisamune et al. (33) revealed that surgical stabilization of multiple rib fractures could shorten mechanical ventilation time and reduce the incidence of pneumonia. Another study focused on rib fractures in the elderly population and showed that mortality was lower in patients who underwent surgery than in patients who received conservative treatment (4% vs. 8%) (34). Therefore, if patients show a novel score >15, the operative treatment could be the optimal treatment option.

In the subgroup analyses, the patients who underwent surgery early achieved better outcomes than those who underwent surgery late in terms of deep vein thrombosis, preoperative pneumonia, combination of chronic disease, and longer hospital stays. Nevertheless, patients with rib fractures often have other injuries, and the whole picture must be considered when making decisions. Surgery should be combined with specific circumstances, and the patient should undergo surgery as soon as possible while remaining stable. Still, the present study does not suggest that surgery must be performed for rib fractures while ignoring the overall safety of the patients. Surgery should be performed as early as possible while ensuring the safety and stability of the patient. The subgroup analyses also showed that as the new score increases, the proportion of rib fracture fixation decreases. A possible explanation could be that the more severe the rib fracture, the more critical the patient's condition, and the surgery may be considered comprehensively to shorten the operation time as much as possible, so only the severely displaced fractures are fixed, resulting in a decrease in the overall fracture fixation ratio. As a result, the variables of tracheal intubation, deep vein thrombosis, hospitalization, return to work/lifetime, and pneumonia were significantly higher in patients with ≤0.75 fixations than in the group with >0.75. This suggests that patients with higher new scores, if conditions permit, should be fully fixed, with at least 0.75 of the fixed rib fractures accounting for the total number of rib fractures.

Currently, the time to return to work or normal life is a widely accepted indicator of functional recovery in trauma patients (35). Although the time to return to work or life was shorter in the surgical group than in the conservative group, the length of hospitalization was significantly longer in the surgical group than in the conservative group, but hospitalization duration was not independently associated with the outcomes. These results suggest that for patients with rib fractures and a novel score of <10, conservative treatment may be more reasonable, as surgery is invasive, expensive, and does not achieve better results. Still, for patients with a novel score ≥10, the incidence of pneumonia, duration of ventilator use, frequency of opioid use, and time to return to work and life were lower in the operated patients than in the conservative group, and the differences were significant (all *P* < 0.05). At all time points during follow-up, the patients in the surgical group were significantly better than those in the conservative group in terms of pain and quality of life, indicating that surgical treatment could be more suitable when the financial condition was acceptable.

This study has several limitations. Firstly, it was a single-center retrospective analysis that relied exclusively on data extracted from patient charts. This reliance introduces potential information and documentation biases, as the chart serves as the sole data source, thereby lacking independent validation mechanisms. Additionally, we did not have access to information regarding patients who sought further examinations or treatments at local hospitals or clinics. The management of each patient was determined by the attending physicians upon admission, which may result in variations and introduce further bias. Secondly, this study represents a preliminary exploration, and the results necessitate validation with external datasets before they can be considered for clinical application. Furthermore, additional research is required to develop a more effective, concise, and user-friendly rib fracture scoring system. Finally, the follow-up period was limited, leaving long-term outcomes undetermined.

## Conclusion

This study validated a novel rib fracture score based on an anatomical and pathophysiological perspective. The novel score showed significant differences among patients with or without pneumonia based on the discriminative performance of the novel score. A cut-off value of 10 was selected. For patients with novel scores <10, surgical treatment did not show enough therapeutic benefits but rather prolonged hospitalization, which may result in a higher healthcare burden. Patients with novel scores ≥10 showed clear benefits from surgical intervention, particularly those with scores ≥15, where continued conservative treatment may result in a significant increase in respiratory-related complications.

## Data Availability

The original contributions presented in the study are included in the article/Supplementary Material, further inquiries can be directed to the corresponding author.
