# Supplementary material for: Clinical application of a novel rib fracture score system in patients with multiple rib fractures
Source: Front Surg. 2026 Jan 8;12:1653221. doi: 10.3389/fsurg.2025.1653221 (PMC12823873; doi:10.3389/fsurg.2025.1653221)
Supplement: Supplementary file 1 [file DataSheet1.docx]

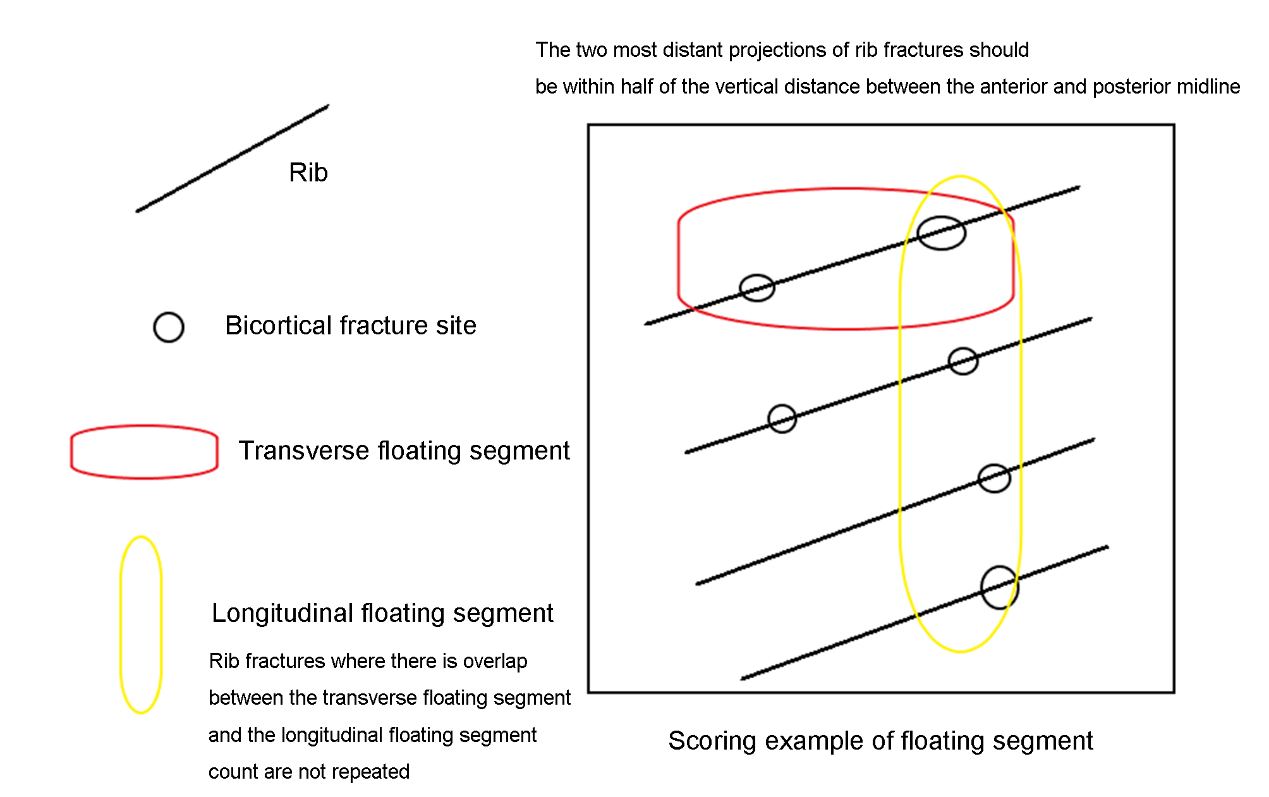


**Supplementary Figure S1.** Schematic diagram of the anatomical scoring system

**Supplementary Table S1.** Pathophysiological score

| Items | Weight |
| --- | --- |
| Pain |  |
| NRS score: 5-6 | 0 |
| NRS score: 7-8 | 1 |
| NRS score: 9-10 | 2 |
| Persistent mandatory protective positions | 3 |
| Hemorrhagic (and/or pneumothorax) | 1 |
| The percentage of lung injury in the total lung volume |  |
| ≤ 18% | 1 |
| 19%-27% | 2 |
| ≥ 28% | 3 |
| Oxygenation status |  |
| Cough and sputum | 1 |
| Unfavorable cough and sputum as judged by a specialist but not yet resulting in the latter condition | 2 |
| A partial pressure of oxygen ≤ 65 mmHg on non-oxygen inhalation | 3 |
| A progressive decrease in finger pulse oximetry or a sustained decrease below 90% | 3 |
| Respiratory rate, times/min |  |
| 21-25 | 1 |
| ≥ 25 | 2 |
| Age, year |  |
| 60-69 | 1 |
| ≥ 70 | 2 |

**Supplementary Table S2.** Univariable and multivariable analysis associated with the occurrence of pneumonia during conservative treatment

| Items | Univariable logistic | | Multivariable logistic | |
| --- | --- | --- | --- | --- |
|  | OR (95% CI) | P | OR (95% CI) | P |
| Age, year | 1.047 (1.030, 1.064) | < 0.001 | 1.151 (1.017, 1.303) | 0.026 |
| Sex |  |  |  |  |
| Male | Ref. |  |  |  |
| Female | 1.093 (0.717, 1.666) | 0.679 |  |  |
| Novel Score | 1.134 (1.096, 1.173) | < 0.001 | 0.988 (0.677, 1.442) | 0.951 |
| Fractures |  |  |  |  |
| Left | Ref. |  | Ref. |  |
| Right | 0.940 (0.598, 1.479) | 0.789 | 0.574 (0.082, 4.006) | 0.576 |
| Bilateral | 2.829 (1.760, 4.547) | < 0.001 | 0.289 (0.019, 4.333) | 0.369 |
| Number of ribs was fixed | 1.397 (1.193, 1.636) | < 0.001 | 1.303 (0.516, 3.292) | 0.575 |
| Number of fixed ribs | 1.218 (1.099, 1.348) | < 0.001 | 0.695 (0.261, 1.851) | 0.466 |
| Other chest injuries and management |  |  |  |  |
| Yes | 1.362 (0.812, 2.285) | 0.241 |  |  |
| No | Ref. |  |  |  |
| Combined chronic diseases, n (%) |  |  |  |  |
| Yes | 2.167 (1.466, 3.201) | < 0.001 | 1.359 (0.177, 10.416) | 0.768 |
| No | Ref. |  | Ref. |  |
| Tracheal intubation, n (%) |  |  |  |  |
| Yes | 5.838 (3.112, 10.952) | < 0.001 |  |  |
| No | Ref. |  |  |  |
| Tracheotomy, n (%) |  |  |  |  |
| Yes | - | 0.999 |  |  |
| No | Ref. |  |  |  |
| ICU duration, hours | 1.019 (1.011, 1.027) | < 0.001 | 0.969 (0.889, 1.057) | 0.475 |
| Deep vein thrombosis, n (%) |  |  |  |  |
| Yes | 12.315 (4.906, 30.911) | < 0.001 | 1.649 (0.136, 20.048) | 0.695 |
| No | Ref. |  | Ref. |  |
| Ventilator use, hours | 1.064 (1.025, 1.104) | 0.001 |  |  |
| Opioid use, times | 3.193 (2.489, 4.096) | < 0.001 | 1.361 (0.346, 5.353) | 0.659 |
| Hospitalization, days | 1.098 (1.073, 1.124) | < 0.001 | 1.269 (1.023, 1.573) | 0.030 |
| Return to work/lifetime, weeks | 1.504 (1.346, 1.681) | < 0.001 | 1.686 (0.722, 3.936) | 0.228 |
| Cost bearer, n (%) |  |  |  |  |
| Insurance | Ref. |  |  |  |
| Self-financed/others | 3.447 (2.044, 5.815) | < 0.001 | 5.331 (0.384, 74.082) | 0.213 |

**Supplementary Table S3.** Univariable and multivariable analysis associated with occurrence of new postoperative pneumonia in surgical patients

| Items | Univariable logistic | | Multivariable logistic | |
| --- | --- | --- | --- | --- |
|  | OR (95%CI) | P | OR (95%CI) | P |
| Age, year | 1.031 (1.003, 1.060) | 0.027 | 1.159 (1.022, 1.315) | 0.022 |
| Sex |  |  |  |  |
| Male | Ref. |  |  |  |
| Female | 1.250 (0.652, 2.396) | 0.502 |  |  |
| Novel Score | 1.150 (1.089, 1.214) | < 0.001 | 1.011 (0.599, 1.707) | 0.967 |
| Flail segment |  |  |  |  |
| No | Ref. |  |  |  |
| Left | 1.282 (0.487, 3.377) | 0.615 |  |  |
| Right | 2.057 (0.934, 4.531) | 0.074 |  |  |
| Bilateral | 2.244 (0.199, 25.341) | 0.513 |  |  |
| Fractures |  |  |  |  |
| Left | Ref. |  | Ref. |  |
| Right | 0.671 (0.326, 1.379) | 0.277 | 1.257 (0.119, 13.298) | 0.850 |
| Bilateral | 2.458 (1.224, 4.933) | 0.011 | 0.843 (0.042, 16.945) | 0.911 |
| Number of ribs was fixed | 1.397 (1.193, 1.636) | < 0.001 | 1.196 (0.479, 2.984) | 0.702 |
| Number of fixed ribs | 1.218 (1.099, 1.348) | < 0.001 | 0.860 (0.292, 2.534) | 0.784 |
| Other chest injuries and management |  |  |  |  |
| Yes | 1.347 (0.701, 2.590) | 0.372 |  |  |
| No | Ref. |  |  |  |
| Combined chronic diseases, N (%) |  |  |  |  |
| Yes | 1.755 (0.981, 3.139) | 0.058 |  |  |
| No | Ref. |  |  |  |
| Tracheal intubation, N (%) |  |  |  |  |
| Yes | 4.909 (2.238, 10.770) | < 0.001 |  |  |
| No |  |  |  |  |
| Ventilator use, hours | 1.061 (1.018, 1.107) | 0.005 |  |  |
| ICU duration, hours | 1.017 (1.009, 1.026) | < 0.001 |  |  |
| Hospitalization, days | 1.139 (1.090, 1.189) | < 0.001 | 1.332 (0.957, 1.856) | 0.090 |
| Return to work/lifetime, weeks | 2.192 (1.457, 3.298) | < 0.001 | 2.543 (0.799, 8.086) | 0.114 |
| Opioid use, times | 3.871 (2.569, 5.833) | < 0.001 | 0.684 (0.120, 3.914) | 0.670 |
| Cost bearer, n (%) |  |  |  |  |
| Insurance | Ref. |  | Ref. |  |
| Self-financed/others | 9.890 (3.001, 32.596) | < 0.001 | 2.701 (0.149, 48.955) | 0.502 |
| Pain Score |  |  |  |  |
| 24 h after surgery | 1.309 (0.829, 2.067) | 0.248 |  |  |
| 72 h after surgery | 1.680 (1.050, 2.686) | 0.030 | 0.084 (0.002, 3.182) | 0.182 |
| 2 weeks after surgery | 1.224 (0.774, 1.937) | 0.388 |  |  |
| 4 weeks after surgery | 1.761 (1.086, 2.858) | 0.022 | 1.003 (0.022, 45.926) | 0.999 |
| 6 weeks after surgery | 1.745 (1.049, 2.903) | 0.032 | 0.981 (0.051, 18.794) | 0.990 |
| 3 months after surgery | 3.848 (2.360, 6.274) | < 0.001 | 5.210 (0.173, 157.220) | 0.342 |
| 6 months after surgery | 6.224 (3.332, 11.626) | < 0.001 | 2.244 (0.064, 78.134) | 0.655 |
| 9 months after surgery | 5.358 (1.656, 17.343) | 0.005 | 0.163 (0.002, 13.463) | 0.421 |

**Supplementary Table S4.** The relationship between operative time and other characteristics

|  | Duration of preoperative conservative treatment ≤ 70 hours (n=155) | Duration of preoperative conservative treatment >70 hours (n=135) | P |
| --- | --- | --- | --- |
| Age, year | 51.41±10.70 | 52.30±11.16 | 0.492 |
| Sex |  |  | 0.177 |
| Male | 123 (79.35) | 98 (72.59) |  |
| Female | 32 (20.65) | 37 (27.41) |  |
| Novel Score | 14 (11, 18) | 15 (11, 20) | 0.259 |
| Deep vein thrombosis, n (%) |  |  | 0.024 |
| Yes | 5 (3.23) | 13 (9.63) |  |
| No | 150 (96.77) | 122 (90.37) |  |
| Flail segment |  |  | 0.911 |
| No | 121 (78.06) | 104 (77.04) |  |
| Left | 13 (8.39) | 14 (10.37) |  |
| Right | 19 (12.26) | 16 (11.85) |  |
| Bilateral | 2 (1.29) | 1 (0.74) |  |
| Other chest injuries and management |  |  | 0.523 |
| Yes | 33 (21.29) | 33 (24.44) |  |
| No | 122 (78.71) | 102 (75.56) |  |
| Preoperative pneumonia |  |  | <0.001 |
| Yes | 2 (1.29) | 57 (42.22) |  |
| No | 153 (98.71) | 78 (57.78) |  |
| Postoperative pneumonia |  |  | 0.125 |
| Yes | 20 (12.90) | 10 (7.41) |  |
| No | 135 (87.10) | 125 (92.59) |  |
| Length of stay | 10 (9, 14) | 14 (11, 20) | < 0.001 |
| Preoperative ICU duration | 0 (0, 0) | 0 (0, 0) | 0.022 |
| Postoperative ICU duration | 0 (0, 0) | 0 (0, 0) | 0.361 |
| Combined chronic diseases |  |  | 0.036 |
| Yes | 46 (29.68) | 56 (41.48) |  |
| No | 109 (70.32) | 79 (58.52) |  |
| Tracheal intubation |  |  | 0.689 |
| Yes | 15 (9.68) | 15 (11.11) |  |
| No | 140 (90.32) | 120 (88.89) |  |
| Tracheotomy |  |  | 0.466* |
| Yes | 0 | 1 (0.74) |  |
| No | 155 (100.00) | 134 (99.26) |  |
| Ventilator use before rib fixation, h | 0 (0, 0) | 0 (0, 0) | 0.040 |
| Ventilator use after rib fixation, h | 0 (0, 0) | 0 (0, 0) | 0.184 |

***Fisher's test**

**Supplementary Table S5.** The relationship between fixed/total rib fractures and other characteristics

|  | Number of fixed/total rib fractures ≤ 0.75 (n=166) | Number of fixed/total rib fractures > 0.75 (n=124) | P |
| --- | --- | --- | --- |
| Novel Score | 11 (14, 18) | 9 (12, 15) | < 0.001 |
| Tracheal intubation, n (%) |  |  | 0.002 |
| Yes | 25 (15.06) | 5 (4.03) |  |
| No | 141 (84.94) | 119 (95.97) |  |
| Deep vein thrombosis, n (%) | |  | 0.021 |
| Yes | 15 (9.04) | 3 (2.42) |  |
| No | 151 (90.96) | 121 (97.58) |  |
| Ventilator use, hours | 0 (0, 0) | 0 (0, 0) | 0.067 |
| ICU duration, hours | 0 (0, 0) | 0 (0, 0) | 0.002 |
| Opioid use, times | 0 (0, 0) | 0 (0, 0) | 0.001 |
| Hospitalization, days | 10 (11, 14) | 9 (10, 12) | <0.001 |
| Return to work/lifetime, weeks | 10 (12, 12) | 9.75 (10, 12) | <0.001 |
| Pain score |  |  |  |
| 24 h after surgery | 3 (4, 4) | 3 (4, 4) | 0.002 |
| 72 h after surgery | 3 (4, 4) | 3 (4, 4) | 0.002 |
| 2 weeks after surgery | 2 (3, 3) | 2 (3, 3) | 0.005 |
| 4 weeks after surgery | 2 (2, 3) | 2 (2, 3) | 0.006 |
| 6 weeks after surgery | 1 (2, 2) | 1 (2, 2) | 0.001 |
| 3 months after surgery | 0 (0, 1) | 0 (0, 0) | <0.001 |
| 6 months after surgery | 0 (0, 0) | 0 (0, 0) | <0.001 |
| 9 months after surgery | 0 (0, 0) | 0 (0, 0) | 0.061 |
| Pneumonia |  |  | 0.002 |
| Yes | 25 (15.06) | 5 (4.03) |  |
| No | 141 (84.94) | 119 (95.97) |  |

Opioid use, times: every 100mg of bucinazine hydrochloride was recorded as one time. Pethidine every 50mg is recorded as 1 time, for example: Qiantongding 200mg, pethidine 100mg=4 times.

**Supplementary Table S6.** The relationship between fixed / total rib fractures sites and other characteristics

| Items | Number of fixed sites/total number of rib fracture sites <=0.75 (n=154) | Number of fixed sites/total number of rib fracture sites >0.75 (n=134) | P |
| --- | --- | --- | --- |
| Novel Score | 14 (10.5,17) | 12 (9,16) | 0.002 |
| Tracheal intubation, n (%) |  |  | < 0.001 |
| Yes | 25 (16.23) | 5 (3.73) |  |
| No | 129 (83.77) | 129 (96.27) |  |
| Deep vein thrombosis, n (%) | |  | 0.003 |
| Yes | 15 (93.74) | 2 (1.49) |  |
| No | 139 (90.26) | 132 (98.51) |  |
| Ventilator use, hours | 0 (0,0) | 0 (0,0) | 0.008 |
| ICU duration, hours | 0 (0,0) | 0 (0,0) | < 0.001 |
| Opioid use, times | 0 (0,0) | 0 (0,0) | 0.065 |
| Hospitalization, days | 11 (10,13.5) | 10 (9,12) | < 0.001 |
| Return to work/lifetime, weeks | 12 (10,12) | 11 (10,12) | 0.001 |
| Pain Score |  |  |  |
| 24 h after surgery | 4 (3,4) | 4 (3,4) | 0.056 |
| 72 h after surgery | 3 (3,4) | 3 (3,4) | 0.059 |
| 2 weeks after surgery | 3 (2,3) | 3 (2,3) | 0.077 |
| 4 weeks after surgery | 2 (2,3) | 2 (2,3) | 0.198 |
| 6 weeks after surgery | 2 (1,2) | 2 (1,2) | 0.090 |
| 3 months after surgery | 0 (0,1) | 0 (0,0) | <0.001 |
| 6 months after surgery | 0 (0,0) | 0 (0,0) | <0.001 |
| 9 months after surgery | 0 (0,0) | 0 (0,0) | 0.085 |
| Pneumonia |  |  | 0.011 |
| Yes | 22 (14.29) | 7 (5.22) |  |
| No | 132 (85.71) | 127 (94.78) |  |
